# Prophylactic Effect of Microwave Radiation on *Toxoplasma gondii* Tachyzoites of RH Strain: A Method for Partial Immunization in BALB/c Mice

**DOI:** 10.1155/japr/1666892

**Published:** 2025-05-27

**Authors:** Amin Shamsaei, Iraj Mohammadpour, Zahra Mohammadi, Qasem Asgari

**Affiliations:** Department of Parasitology and Mycology, School of Medicine, Shiraz University of Medical Sciences, Shiraz, Iran

**Keywords:** flow cytometry, infectivity, MAT, microwave, survivability, *Toxoplasma gondii*

## Abstract

*Toxoplasma gondii* is a ubiquitous protozoan parasite causing toxoplasmosis in humans. The goal of this study was to examine the effect of microwave on the viability and infectivity of *T. gondii* tachyzoites of the RH strain, both in vitro and in vivo. *T. gondii* tachyzoites of the RH strain were treated with microwave radiation for 5, 10, 15, and 20 s. The viability of treated tachyzoites was assessed using flow cytometry. The in vitro infectivity of these treated tachyzoites was investigated using the HeLa cell culture. For in vivo studies, BALB/c mice received two injections of treated tachyzoites mixed with Freund's adjuvant, over a fortnight interval, and their daily survival rates were monitored. Subsequently, untreated tachyzoites were inoculated into surviving mice in order to evaluate induced immunity. The produced IgG antibody titers in surviving mice were measured using the modified agglutination test. The flow cytometry method showed mortality rates of 46.89%, 74.11%, 88.38%, and 99.34% for the treated tachyzoites at 5, 10, 15, and 20 s, respectively. An in vitro analysis showed no proliferation of treated tachyzoites at 10- to 20-s time points after 24, 48, and 72 h. An in vivo study showed that all mice injected with tachyzoites treated for 5 s died, while those treated for 10–20 s survived. Mice that survived were exposed to untreated tachyzoites and showed a significant viability rate up to 18 days. The modified agglutination test showed an antibody titer of 1:80 in partially immunized mice. These results suggest that microwave-treated tachyzoites combined with Freund's adjuvant greatly enhance survival rates, reduce infectivity, and induce a humoral immune response in mice, offering partial protection against acute toxoplasmosis.

## 1. Introduction

Humans and other warm-blooded animals are infected by the protozoan parasite *Toxoplasma gondii*. One of the most prevalent parasitic diseases worldwide is toxoplasmosis. The risk of contracting toxoplasmosis is around one-third of the global population. It can be transmitted via water, food, soil, and congenitally [1–3]. This parasite has both sexual and asexual reproduction stages in its life cycle. It is a member of the phylum Apicomplexa [4].

In both the veterinary and medical domains, *T. gondii* is extremely important. In domestic animals, it can result in abortion and stillbirth [5, 6]. In humans, it can result in a number of symptoms, such as fever, fatigue, myalgia, and lymphadenitis. Encephalitis, dementia, retinochoroiditis, microphthalmia, hydrocephaly, and microcephaly are all consequences of congenital toxoplasmosis. Infertility, abortion, and neonatal death are further outcomes of maternal infection [7, 8]. In immunocompromised and pregnant women, serious side effects including neurological and vision impairments, miscarriage, and stillbirth are possible outcomes [9–11]. It is imperative to take effective preventive measures against toxoplasmosis due to these significant health impacts.

Currently, pyrimethamine and sulfadiazine are used as part of the standard treatment for toxoplasmosis, which can cause serious adverse effects like kidney failure and bone marrow suppression [12, 13]. Spiramycin is the recommended medication for treating toxoplasmosis in pregnant women; however, it comes with side effects, including urticaria, skin rashes, itching, jaundice, and bleeding in the gastrointestinal tract [14]. Nonetheless, the latent form of the disease and tissue cysts are not treated by these medications [15].

Developing novel, potent vaccines is one strategy to counteract the different clinical consequences of toxoplasmosis and can be a powerful instrument in the advancement of world health. In addition to minimizing drug side effects, vaccines are highly effective tools for immunization and disease prevention. Inactivated and attenuated live vaccines, as well as DNA, protein, and epitope approaches, have all been employed in the development of toxoplasmosis vaccines [16–19]. Currently, Toxovax, the only toxoplasmosis vaccine available commercially, is based on the S48 strain. However, it is possible for this vaccine to revert to the virulent phenotype, because of its short half-life and inability to totally prevent vertical transmission [20].

To date, *T. gondii* oocysts have been rendered inactive by a variety of techniques, including chlorine, ozone and ultraviolet (UV) radiation, radiofrequency waves, gamma irradiation, and low-energy electron irradiation [21–26]. Establishing immunity through safe, effective, and affordable methods is the best preventive strategy for toxoplasmosis because of its high importance as a health problem and the possibility of transmission to susceptible populations.

Microwaves are extremely high-frequency electromagnetic waves with short wavelengths. Their wavelength falls in between the infrared and radio spectrums. These waves have frequencies in the range of 300 MHz–300 GHz. Only a straight line (the line of sight) can transmit microwaves. The energy required for chemical changes through ionization is not present in these waves [27]. Microwaves are used in a wide range of industries and fields, including spectroscopy, wireless networks, mobile phones, point-to-point communications, radars, plasma engineering applications, and ovens [28].

Moreover, microwave irradiation has been demonstrated to have potential practice in various biomedical applications, such as pathogen inactivation and sterilization. Microwave irradiation has the ability to quickly and uniformly heat biological samples, resulting in the denaturation of proteins and nucleic acids without much deterioration of structural components. This characteristic makes it a promising option, because it has the potential to deactivate disease-causing organisms while keeping the important antigenic features needed for immune system detection. Prior studies have shown that microwave radiation effectively kills bacteria, viruses, fungi, and parasites [29, 30]. Thus, the purpose of this study was to examine the immunization against RH strain tachyzoites of *T. gondii* using microwave exposure in BALB/c mice. As far as we know, this is the first investigation into how microwave affects RH strain tachyzoites of *T. gondii*.

## 2. Materials and Methods

### 2.1. Ethics Statement

All animal experiments and care procedures were organized under the instructions and endorsement of the Institutional Animal Care Committee (IACC) of Shiraz University of Medical Sciences (IR.SUMS.AEC.1402.058) for animal ethical and welfare standards. The experiments were carried out on BALB/c mice obtained from the Animal Laboratory of Shiraz University of Medical Sciences.

### 2.2. Parasite Preparation

The Tehran University of Medical Sciences, located in Tehran, Iran, provided the RH strain of *T. gondii*. Female BALB/c mice (5–6 months old, weighing 25–30 g) were inoculated intraperitoneally with a suspension of the RH strain (0.2 mL, corresponding to 1 × 10^5^ tachyzoites) using insulin syringes, and the mice were euthanized after 96 h. Subsequently, tachyzoites were obtained from the peritoneal cavity of infected mice by rinsing with 5 mL of sterile phosphate-buffered saline (PBS) and were centrifuged at 200 × *g* for 10 min at 25°C. The pellets with tachyzoites were retrieved using PBS, and 0.2 mL of a 1 × 10^5^/mL suspension was inoculated intraperitoneally into more mice. Flow cytometry was employed to evaluate the viability of tachyzoites and their ability to penetrate HeLa cells.

### 2.3. In Vitro Assay

#### 2.3.1. Microwave Treatment

After preparing a cellular suspension (1 × 10^5^ tachyzoites/1 mL of PBS), the tubes were tightly closed and exposed to a household Panasonic microwave oven (Kadoma, Osaka, Japan) with a maximum power of 800 W and a constant frequency of 2450 MHz for 5, 10, 15, and 20 s to evaluate the effect of microwave on *T. gondii* tachyzoites. The oven featured a rotating dish to ensure optimal microwave distribution inside the tubes, and the samples were rotated during the treatment process. All experiments for the different time conditions were conducted in triplicate.

#### 2.3.2. Flow Cytometric Assessments

Flow cytometry using propidium iodide (Sigma-Aldrich, Sofia, Bulgaria) staining was used to evaluate the viability of tachyzoites exposed to microwave radiation. Tachyzoites treated with 0.2% saponin and untreated tachyzoites were used as positive and negative controls, respectively. Each tube was filled with 10 *μ*L of propidium iodide staining solution (50 *μ*g/mL), and they were incubated for 20 min at 4°C in the dark. The staining mechanism depends on dead or apoptotic cells' nuclei absorbing propidium iodide. After that, the mixtures were put into polystyrene flow cytometry tubes. The flow cytometer was set up using untreated tachyzoites, and gating was done in accordance with the unique size (2 × 6*  μ*m) and distribution of the tachyzoites. Fluorescence minus one served as a control for gating. Cells were run on a BD FACSCalibur analytical flow cytometer, equipped with a 15-mV, 488-nm air-cooled argon ion laser (Becton–Dickinson, San Jose, California, United States). Multiparametric data were analyzed using the FlowJo software (Ashland, Oregon, United States). The device was set to detect propidium iodide, and 10,000 cells/tube were counted. All experiments were executed in a specific laboratory, and the equipment was disinfected with FACS-safe solution (1% active chlorine; Becton–Dickinson).

#### 2.3.3. Assay for Extracellular Treated Tachyzoites

The HeLa cells were cultured in RPMI-1640 medium (Biowest, Bradenton, Florida, United States) supplemented with 15% heat-inactivated fetal bovine serum (Zen-Bio, Durham, North Carolina, United States), 2 mM l-glutamine, 100 U/mL penicillin, and 100 *μ*g/mL streptomycin (Biocompare, San Francisco, California, United States). The trypan blue (Sigma-Aldrich, Wien, Austria) staining method was used to calculate the proportion of viable HeLa cells. In this technique, dead cells absorb the dye, whereas living cells repel it.

The microwave-treated tachyzoites were used to assess their invasion of HeLa cells by being introduced to the wells at a 5:1 infection ratio (parasites:cells) and then placed in an incubator at 37°C in 5% CO_2_. The cells were detached and then centrifuged for 3 min at 500 × *g* after 24, 48, and 72 h. Following that, the pellets were spread out and stained with Giemsa (Merck, Darmstadt, Germany). The growth, reproduction, and parasite load (number of infected cells × average number of tachyzoites within infected cells/100 HeLa cells) were recorded.

### 2.4. In Vivo Assay

Sixty BALB/c mice were divided into six groups. Subsequently, the microwave-treated tachyzoites were suspended in PBS (0.2 mL, corresponding to 1 × 10^5^ tachyzoites) mixed with Freund's complete adjuvant (Sigma-Aldrich, Saint Louis, Missouri, United States) to form an emulsion, which was then subcutaneously injected into mice. For the positive control group, 10 mice were injected with PBS containing untreated tachyzoites mixed with Freund's complete adjuvant. For the negative control group, 10 mice were injected with PBS mixed with Freund's complete adjuvant. Acetaminophen (Sigma-Aldrich, Wien, Austria, 1–2 mg/mL drinking water) was administered for analgesic and sedative effects. The mice were monitored daily. Two weeks later, blood was collected from the surviving mice for the MAT testing. After cleaning the collection site with 70% ethanol, each mouse was held still while 0.2 mL of blood was drawn from the lateral tail vein using a 25- to 27-gauge needle. Hemostasis was attained by exerting pressure with sterile gauze to halt blood flow. One day following the blood sampling, an additional dose containing Freund's incomplete adjuvant was given via subcutaneous administration. Two weeks later, blood was collected from the surviving mice. One day after the second blood sampling, mice were injected with PBS containing untreated tachyzoites to evaluate immunity. The mice were observed every day until they passed away. The brain, spleen, and blood smears from dead mice were made and stained with Giemsa to detect the cause of death.

To explore partial immunization in mice, a 0.2 mL suspension of a 1 × 10^5^ intact tachyzoites/mL sterile PBS solution was inoculated subcutaneously into the surviving mice, and their survival was observed.

### 2.5. Modified Agglutination Test

The MAT was used to determine specific IgG antibodies to *T. gondii* in mice sera, as previously explained [31, 32]. An antigen made up of *T. gondii* RH strain tachyzoites extracted from the peritoneal cavity of mice was utilized. Tachyzoites that were purified were treated with 6% formalin and left to store at 4°C for one night. Following fixation, the formalin suspension underwent centrifugation, was washed three times in sterile PBS, and then resuspended in alkaline borate buffer (pH 8.7) with 0.4% bovine serum albumin (BSA/borate buffer) and 0.2% sodium azide to reach a final concentration of around 2 × 10^8^/mL (antigen stock suspension). It was kept at 4°C until needed. The antigen mixture for each plate was created by combining 200 *μ*L of tachyzoites fixed with formalin, 2.5 mL of BSA/borate buffer, 35 *μ*L of 2-mercaptoethanol, and 50 *μ*L of Evans blue dye solution (2 mg/mL water). Equal volumes (25 *μ*L) of newly made antigen mixture and diluted sera were added to each well and gently mixed by pipetting repeatedly. Sera from mice were diluted in increments of two starting from 1:20 to 1:2560 and then tested. Antibody titers equal to or above 1:20 were considered positive. Both positive and negative sera were included in every plate at the same dilutions as the test sera to act as controls. The plate was sealed with tape, left at room temperature, and readings taken after 24 h. A blue pellet in the base of the “U” bottom 96-well microplates (Greiner Bio-One, Kremsmünster, Austria) indicated a negative result, while a clear bottom indicated a positive result.

### 2.6. Statistical Analysis

The log-rank (Mantel–Cox) and Kaplan–Meier tests were utilized to examine correlations among survival curves. Statistical analyses were conducted using SPSS (SPSS 27.0, Chicago, Illinois, United States), and a *p* value < 0.05 was considered significant.

## 3. Results

### 3.1. Flow Cytometric Measurements

**Figure 1:**
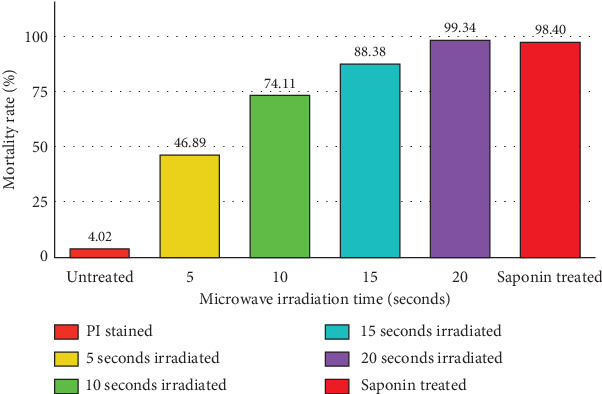
Illustration of the relationship between the mortality rate percentage of treated tachyzoites versus untreated ones and other materials.

**Figure 2:**
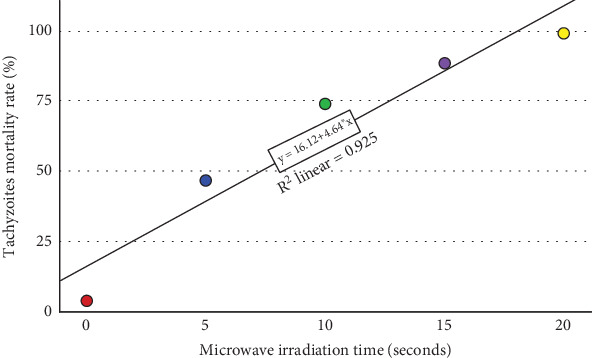
Correlation between the mortality rate and duration of 50% inhibition of treated tachyzoites.

The results of the flow cytometry tests indicated that around 98.1% of the *T. gondii* tachyzoites gathered from the peritoneal fluids of mice were viable. The outcome of the propidium iodide staining for untreated tachyzoites showed that 4.02% of the tachyzoites were dead. The mortality rate of tachyzoites was 98.40% as a result of being exposed to 0.2% saponin during propidium iodide staining. Flow cytometry revealed that the mortality rates of microwave-treated tachyzoites at a maximum power of 800 W and a constant frequency of 2450 MHz for 5, 10, 15, and 20 s were 46.89%, 74.11%, 88.38%, and 99.34%, respectively (Figure 1). Accordingly, the duration of 50% inhibition of treated tachyzoites was calculated to be 7.3 s (Figure 2). Flow cytometry histogram plots are presented in Figure S1.

### 3.2. Microwave Treatment Results

#### 3.2.1. In Vitro

The negative control group utilized pure HeLa cells, while the positive control group employed HeLa cells along with intact tachyzoites. No tachyzoite replication or growth was detected in HeLa cells after 24, 48, and 72 h in the groups that were subjected to microwave radiation for 10, 15, and 20 s. After 24 h, the parasite load for positive control group was 26.5%. In contrast, the group that was subjected to 5 s of microwave radiation experienced a 15.8% parasite load.

#### 3.2.2. In Vivo

**Figure 3:**
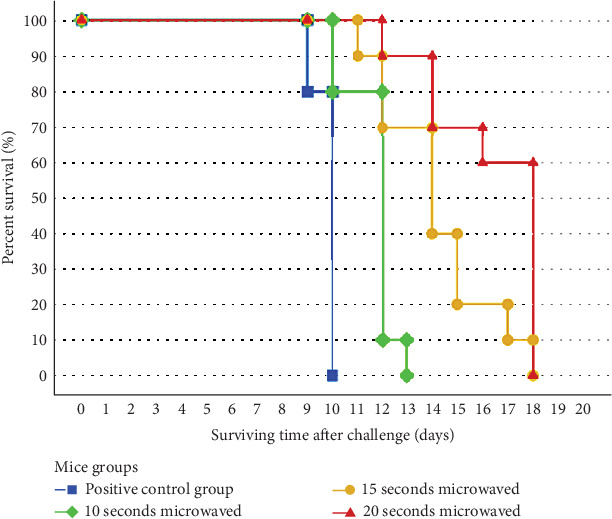
Exhibition of survival times of Groups 2, 3, and 4 compared to the positive control group.

The positive control group included mice that were injected with intact tachyzoites, whereas the negative control group did not receive any tachyzoite injections. Groups 1–4 of mice received injections of microwave-treated tachyzoites lasting 5–20 s each. The group of mice that received treatment for 20 s exhibited the longest average survival time (16.40 ± 2.27 days, *p* < 0.001), whereas the positive control group demonstrated the shortest (10.40 ± 0.52 days) compared to the other groups. Mice that received tachyzoites exposed to 5 s of microwave radiation lived much longer (13.40 ± 0.70 days, *p* < 0.01) than the positive control group of mice injected with intact tachyzoites. In addition, mice that were injected with tachyzoites exposed to microwave radiation for 10 (13.70 ± 0.95 days, *p* < 0.05) and 15 (14.20 ± 2.20 days, *p* < 0.01) s showed significantly longer survival compared to the positive control group (Figure 3).  Table 1 displays the data validation statistics regarding the overall survival days of mice that were exposed to both microwave-treated and intact tachyzoites in the study groups.

**Figure 4:**
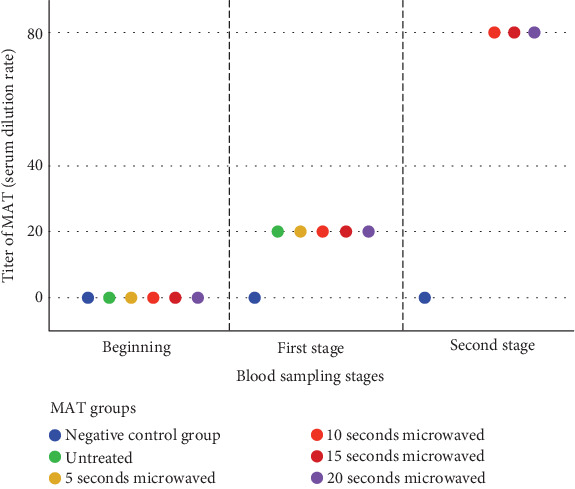
Demonstration of the MAT test among untreated and microwave-treated tachyzoites.

In Week 4, the MAT showed positivity with a titer of 1:80 for Groups 2–4 (10, 15, and 20 s) and negativity for the control group (Figure 4). Mice from Group 1 (5 s) succumbed to toxoplasmosis prior to reaching Week 4.

## 4. Discussion

*T. gondii* is a parasite that is found worldwide. Cats and other felines serve as the definitive hosts, with warm-blooded animals like mammals and birds functioning as intermediate hosts [33, 34]. In immunocompetent people, symptoms are typically mild and manifest as fever, lymphadenitis, fatigue, and myalgia [7]. Congenital transmission may lead to issues like fetal abortion, chorioretinitis, hydrocephaly, and microcephaly in infants [8]. In individuals with HIV, the infection has the potential to be deadly, frequently resulting in encephalitis [9].

The common pharmacological therapy for toxoplasmosis includes the simultaneous administration of pyrimethamine and sulfadiazine, which can cause harmful side effects such as bone marrow suppression and renal failure [12, 13]. At present, the only vaccine on the market is Toxovax, which is not meant for human vaccination [20]. Recent attempts to create toxoplasmosis vaccines have concentrated on creating weakened live strains of *T. gondii*, through the deletion of important genes like GRA17 from the RH strain and CDPK2 from the PRU strain [35, 36]. The RH strain of *T. gondii*, with a deleted GRA17 gene, can trigger a protective immune response against infection and diminish tissue cysts in the brains of vaccinated mice [35]. Moreover, knocking out the tyrosine kinase gene prevents *T. gondii* from binding to host cells, stops the lytic cycle, and hinders disease progression in mice [37].

Microwaves, which fall within the nonionizing radiofrequency range of electromagnetic radiation, have significant impacts on living organisms. Aside from the heat generated, microwaves also induce nonthermal (resonance) effects in living organisms [28]. The impact of microwaves that are not related to heat is most noticeable at the organism and, to a smaller degree, at the cellular levels, with limited effects seen at the molecular level. The reason for this is that electromagnetic fields influence living tissue both energetically and informatively [28].

Water is crucial in the absorption of microwave energy by living things. It plays a significant role in the dielectric losses of microwave energy in biological tissue. The thermal effect of electromagnetic induction is primarily influenced by the relaxation of dipolar water molecules and the oscillation of free charges in a microwave field. Heating organism cells and tissues leads to alterations in the physicochemical characteristics of biological structures [29, 30]. The impact of microwave radiation on living things depends on the level of electromagnetic energy that is able to penetrate and be absorbed by them. A considerable amount of microwave energy is transformed into heat, causing the internal organs to be destroyed and potentially leading to the parasite's body rupturing due to the ions and water molecules in tissues oscillating [29, 30].

In our study, we examined the impact of microwave radiation on *T. gondii* tachyzoites for the first time. The findings indicated that subjecting tachyzoites to 2450 MHz microwave radiation for 10, 15, and 20 s resulted in higher mortality rates and decreased parasite load in HeLa cells. The inactivation of *T. gondii* tachyzoites by microwave at a constant frequency seems to depend on the exposure time.

So far, different methods have been used to deactivate *T. gondii* oocysts, such as chlorine, ozone, UV radiation, radiofrequency waves, gamma irradiation, and low-energy electron irradiation [21–26]. A 2018 study in China assessed how the combination of UV radiation and disodium cromoglycate (DSCG) boosts immunity against *T. gondii*. The research showed that UV radiation might influence the immune response, and the effectiveness could be enhanced by adding DSCG [38]. A different study conducted in 2013 found that 1-min exposure to UV radiation inhibited the growth of *T. gondii* tachyzoites and formation of tissue cysts without impacting the host's humoral immune response [39]. In another study conducted in Brazil, the *T. gondii* antigen protein was isolated and tested for its vulnerability to gamma radiation. The study discovered that 1500 Gy was the best dose to change the structure of the *T. gondii* protein [25]. In another study, the same team found that immunization with gamma-irradiated *T. gondii* tachyzoites induced a permanent humoral immune response in the intraperitoneally immunized mice, with a significant increase in the production of specific high-affinity IgG antibodies after 120 days of immunization compared with the group without immunization [40].

Another research examined the effectiveness of sodium hypochlorite and ozone in inactivating *T. gondii* oocysts in water. The findings indicated that *T. gondii* could not be inactivated by these two compounds, even at elevated levels [21]. In another study conducted by the same team, they evaluated the impact of radiofrequency waves on *T. gondii* oocysts present in water. The findings indicated that a 1-min exposure to radiofrequency could deactivate the oocysts and protect mice from infection by utilizing the generated heat [23]. In a study managed in 2023, the hybrid formaldehyde-killed tachyzoites of *T. gondii* RH and GT1 isolates with aluminum adjuvant induced humoral and cellular immune responses of BALB/c mice and offered mildly protective efficacy against acute toxoplasmosis [41]. Another study conducted in 2023 showed that low-energy electron irradiation-attenuated *T. gondii* tachyzoites induced high levels of antibodies and protected the animals from acute infection [26]. A systematic review study assessed various techniques for inactivating *T. gondii* oocysts. Of the 309 methods assessed, 110 (35.60%) were successful in deactivating the oocysts. Physical techniques proved to be more efficient than other methods. Disinfectants are not as effective in inactivating *T. gondii* oocysts when used in water. Radiation and pressure were discovered to efficiently deactivate oocysts, and because these methods do not require temperature alterations, they are suitable for treating food items like meat and vegetables without causing notable changes in their physical and chemical characteristics. Thus, these techniques could be viewed as suitable options for deactivating *T. gondii* oocysts [42].

A research conducted in 2008 assessed the immune response induced by a DNA vaccine with *T. gondii* SAG1, ROP2, and GRA2 antigens in BALB/c mice. The findings showed that combining this vaccine with IL-12 as an adjuvant greatly boosts the immune response and extends the lifespan of mice [43]. Another study examined the immune response of C57BL/6 mice to a combination of recombinant GRA2 and GRA6 antigens of *T. gondii* with monophosphoryl lipid A (MPL) adjuvant. The findings indicated that this mixture could trigger a certain level of protection in mice, but there is ongoing discussion about the effectiveness and duration of these antigens when used alone as a vaccine [44]. Furthermore, live attenuated mutant forms of *T. gondii* create powerful immunity against toxoplasmosis in mice and cats [45, 46].

A 2007 study assessed the efficiency of ultrasound in deactivating *Cryptosporidium parvum* oocysts in water. The research showed that exposing *Cryptosporidium* oocysts to sonication at 1 MHz for 20 min effectively inactivated them [47]. Another research assessed the effectiveness of ozone, ultrasound, and a combination of both methods in removing *Giardia* cysts from wastewater. The findings indicated that employing ozone, ultrasound, or a combination of both resulted in a notable reduction in the quantity of *Giardia* cysts [48]. In addition, another team of researchers studied the impact of 375 kHz ultrasound with 24.4 W power for 10, 20, and 40 min on *Giardia* cysts in water. Findings indicated that employing this method for a duration of 20 min led to a 52.4% decrease in *Giardia* cysts [49].

Moreover, multiple research studies have shown that microwave radiation is able to efficiently inactivate certain types of parasites. In a study managed in 2011, the viability of *Anisakis simplex* (isolated larvae and infected fish muscle) heated in a microwave oven with precise temperature control was compared with that of larvae heated in a water bath to investigate any additional effect of the microwaves. Microwave treatment killed *A. simplex* larvae faster than did conventional cooking when the microwaves fully penetrated the samples and resulted in fewer changes in the fish muscle [50]. A study accomplished in 2016 appraised the immunizing capacity of microwave-irradiated infective larvae of *Strongyloides ratti* in rats. Their results demonstrated that microwaved, infective larvae (intact or homogenized) of *S. ratti* were immunogenic for rats. The immunity elicited by exposure to microwaved larvae was characterized on challenge by a significant reduction in the number of eggs produced/worm, by the formation of perioral plugs, and by reductions in worm numbers and size [51].

Other studies investigated the influence of microwave radiation on the metacercariae of *Opisthorchis felineus* and the larvae of three *Trichinella* sp. The high efficiency of microwave radiation on the viability of these species is shown. Even short-term exposure leads to complete death of the larvae of the helminths in the muscle tissue [52, 53]. In a study managed in 2023, the effect of microwave on malaria parasites was assessed. The results showed that microwave energy killed more than 90% of the *Plasmodium falciparum* parasites, not by a thermal effect but via a microwave energy-induced programmed cell death. The findings presented a molecular insight into the elusive interactions of oscillating electromagnetic fields with *P. falciparum* [54].

## 5. Conclusion

According to our findings, microwave can lead to a significant decrease in *T. gondii* tachyzoites' activity. In the in vitro examination, no replication or growth of tachyzoites was observed in HeLa cells after 24, 48, and 72 h in the groups exposed to microwave radiation for 10, 15, and 20 s. In the in vivo investigation, mice showed a notable increase in survival rate compared to the control group. Moreover, the MAT demonstrated that anti–*T. gondii* IgG antibodies were generated in every mouse that survived. Hence, microwave can offer surface-level disinfection similar to gamma and UV radiations in treating *T. gondii* tachyzoites. Taking into account the notable dangers posed by gamma and UV radiation towards the environment and the potential for inadvertent mutations in the parasite's genetic material, the utilization of microwaves might help decrease these risks. These findings suggest exploring different intervention methods and assessing immunomodulatory effects by measuring cytokines and other immunological factors. These results could help in the advancement of safer and more efficient approaches to manage toxoplasmosis.

## Figures and Tables

**Table 1:** Data validation statistics of the total survival days of mice exposed to microwave-treated and intact tachyzoites.

**Groups**	**Mean** ^**a**^	**SD**	**95% confidence interval (days)**	
			**Lower bound**	**Upper bound**
5 s	13.4	0.70	12.90	13.90
10 s	41.70	0.95	43.02	44.38
15 s	44.20	2.20	44.63	47.77
20 s	46.40	2.27	46.78	50.02
Positive control	10.40	0.52	10.03	10.77
Negative control	60.000^b^	0.929	59.801	60.198

^a^Mean: average survival days of mice in different study groups.

^b^The typical length of time that the mice were observed in the negative control group.

## Data Availability

The data that support the findings of this study are available from the corresponding authors upon reasonable request.

## Supporting Information

Supporting Information Additional supporting information can be found online in the Supporting Information section. Figure S1: Histogram plots of *T. gondii* tachyzoite cells. (a) Negative control group without PI staining, (b) PI staining of untreated, and exposed to (c) 0.2% saponin: (d) 5 s, (e) 10 s, (f) 15 s, and (g) 20 s of microwave radiation.
