# Supplementary figures and images for: Prophylactic Effect of Microwave Radiation on Toxoplasma gondii Tachyzoites of RH Strain: A Method for Partial Immunization in BALB/c Mice
Source: J Parasitol Res. 2025 May 27;2025:1666892. doi: 10.1155/japr/1666892 (PMC12133370; doi:10.1155/japr/1666892)

## Slide 1
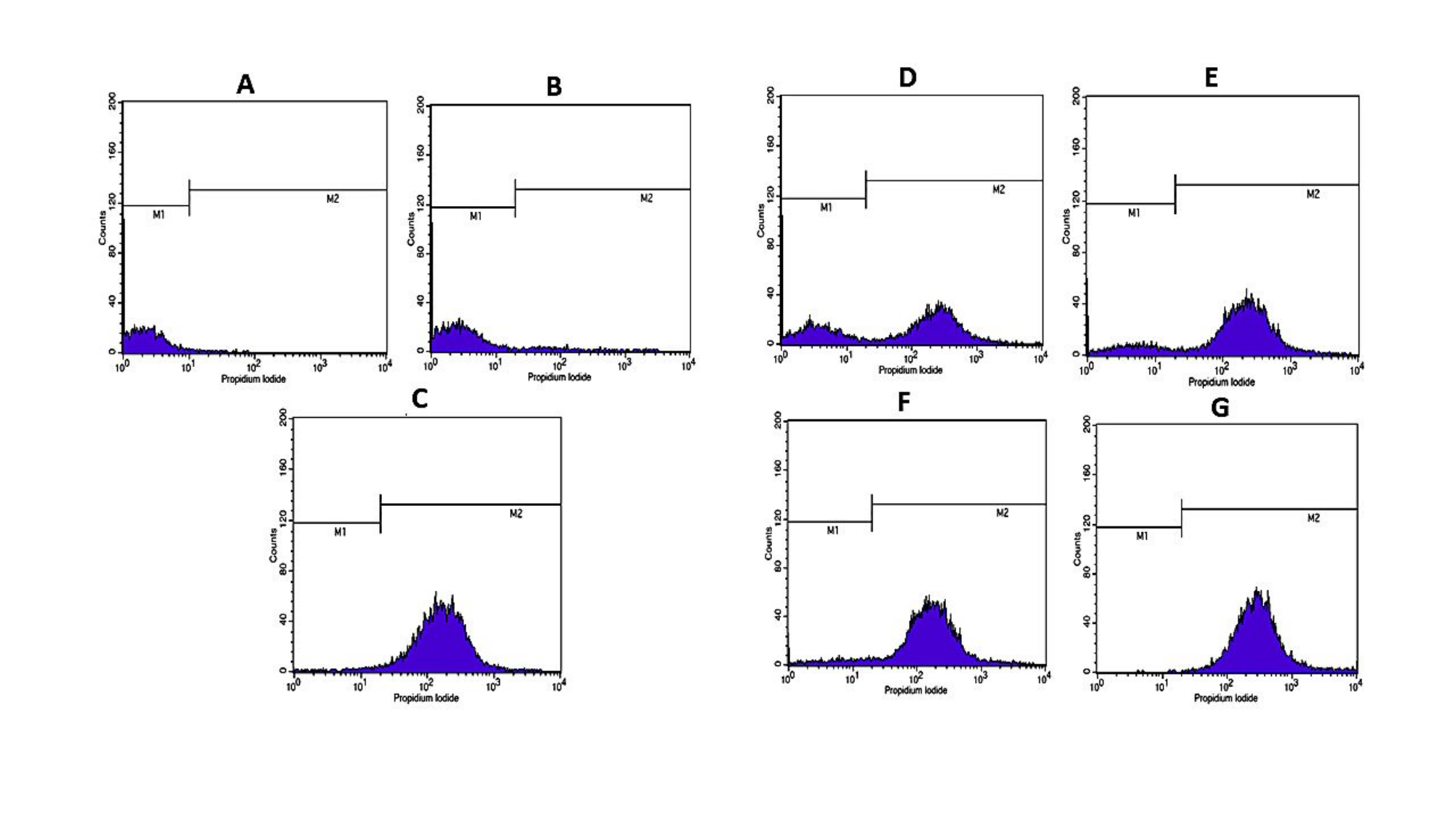

Supplement: Supporting Information — Additional supporting information can be found online in the Supporting Information section. Figure S1: Histogram plots of T. gondii tachyzoite cells. (a) Negative control group without PI staining, (b) PI staining of untreated, and exposed to (c) 0.2% saponin: (d) 5 s, (e) 10 s, (f) 15 s, and (g) 20 s of microwave radiation. [file 1666892.f1.pptx]
